# Frontiers of Complex Disease Mechanisms: Membrane Surface Tension May Link Genotype to Phenotype in Glaucoma

**DOI:** 10.3389/fcell.2018.00032

**Published:** 2018-04-06

**Authors:** Howard R. Petty

**Affiliations:** Department of Ophthalmology and Visual Sciences, The University of Michigan Medical School, Ann Arbor, MI, United States

**Keywords:** surface tension, glaucoma, open-angle, cell membrane, oxidative stress, pressures

## Abstract

Although many monogenic diseases are understood based upon structural changes of gene products, less progress has been made concerning polygenic disease mechanisms. This article presents a new interdisciplinary approach to understand complex diseases, especially their genetic polymorphisms. I focus upon primary open angle glaucoma (POAG). Although elevated intraocular pressure (IOP) and oxidative stress are glaucoma hallmarks, the linkages between these factors and cell death are obscure. Reactive oxygen species (ROS) promote the formation of oxidatively truncated phosphoglycerides (OTP), free fatty acids, lysophosphoglycerides, oxysterols, and other chemical species that promote membrane disruption and decrease membrane surface tension. Several POAG-linked gene polymorphisms identify proteins that manage damaged lipids and/or influence membrane surface tension. POAG-related genes expected to participate in these processes include: *ELOVL5, ABCA1, APOE4, GST, CYP46A1, MYOC*, and *CAV*. POAG-related gene products are expected to influence membrane surface tension, strength, and repair. I propose that heightened IOP overcomes retinal ganglion cell (RGC) membrane compressive strength, weakened by damaged lipid accumulation, to form pores. The ensuing structural failure promotes apoptosis and blindness. The linkage between glaucoma genotype and phenotype is mediated by physical events. Force balancing between the IOP and compressive strength regulates pore nucleation; force balancing between pore line tension and membrane surface tension regulates pore growth. Similar events may contribute to traumatic brain injury, Alzheimer's disease, and macular degeneration.

A major challenge in molecular medicine is to understand polygenic disease mechanisms. In contrast to monogenic diseases exhibiting one aberrant gene, dozens or hundreds of genes influence polygenic diseases. Linking genotype to phenotype is difficult because: genetic effects are small, substantial complexity exists, and multiple pathways yield the same clinical phenotype. This review uses glaucoma as a model polygenic disease. Vision loss in glaucoma is due to retinal ganglion cell (RGC) apoptosis. The hallmarks of primary open angle glaucoma (POAG) are increases in intraocular pressure (IOP) and reactive oxygen species (ROS) production (Kwon et al., 2009; Njie-Mbye et al., 2013). ROS are produced by: phototoxicity, mitochondria, leukocytes, and endogenous oxidases. Oxidative stress may damage the trabecular meshwork and retina. ROS production and activation of the lipid peroxidation pathway (LPP) have been observed in RGC (Ko et al., 2005; Yücel et al., 2005; Chidlow et al., 2017). Products of phospholipid truncation during the LPP have been found in the retina and aqueous humor of glaucoma patients, but not controls (Njie-Mbye et al., 2013). The presence of oxidatively modified retinal proteins has been confirmed (e.g., Tezel et al., 2005). Peroxynitrite, a product of superoxide and nitric oxide, damages RGCs as evidenced by 3-nitrotyrosine protein modification (Neufeld, 1999). However, the mechanism linking IOP, oxidants, and cell death is unclear. This article considers recent findings in the molecular biology of POAG in the context of oxidative biochemistry and membrane biophysics to identify a mechanism. I propose: when the stress of heightened IOP exceeds the compressive strength of an oxidant-damaged RGC membrane, the ensuing structural failure initiates apoptosis. The unifying premise that cellular chemistry couples with external forces via physical properties of RGC surfaces simultaneously explains RGC death, membrane-related genetic polymorphisms, the requirement for oxidative damage, low grade inflammation, mitochondrial defects, and the lack of pathological damage in early disease.

## Lipids

Lipids are heterogeneous amphipathic molecules that self-organize into structures, including membranes, in water. This section focuses on the abundant lipids docosahexaenoic acid (DHA) and cholesterol, whose properties change dramatically upon oxidation.

### DHA

**Figure 1:**
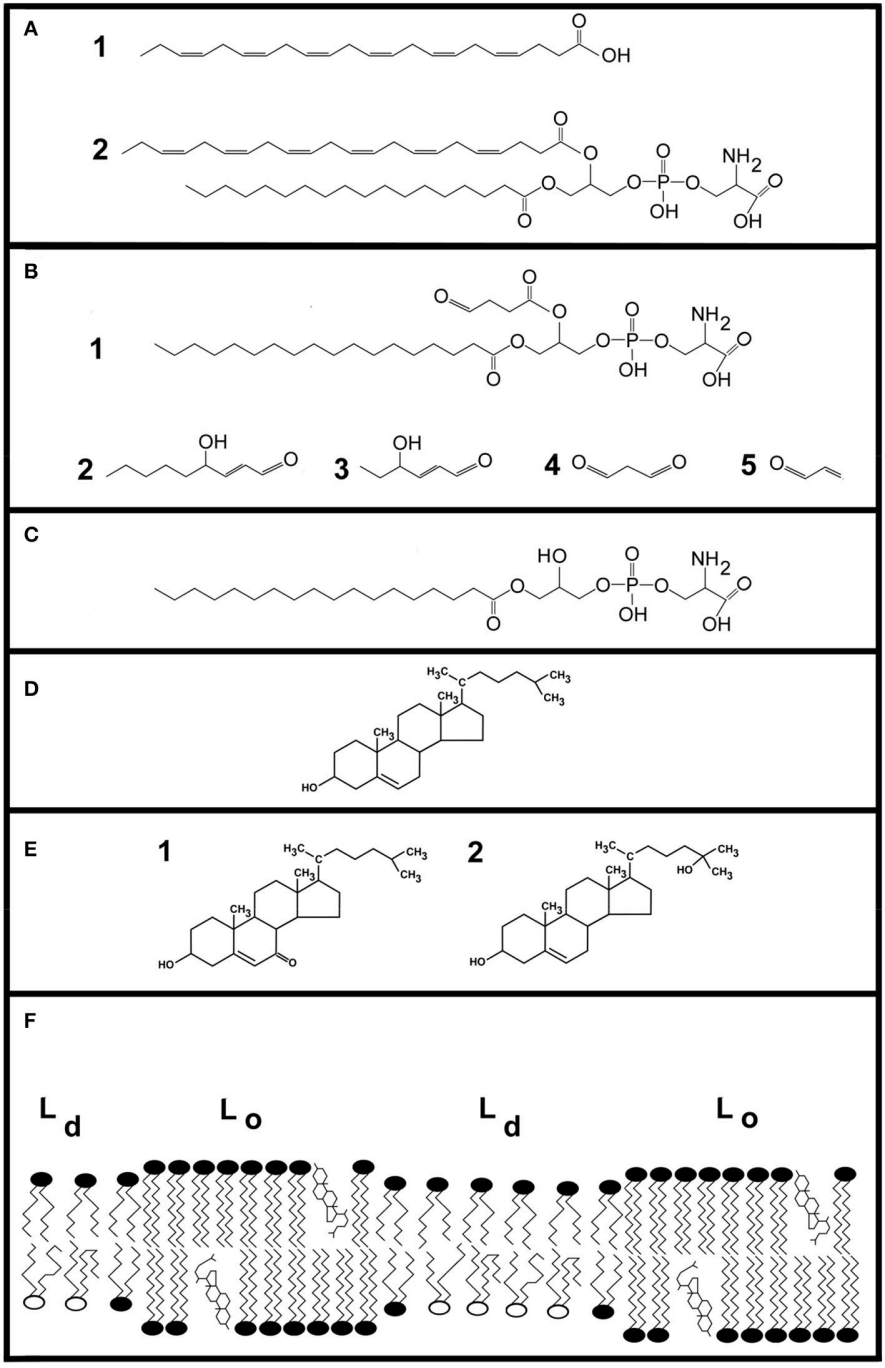
Illustrations of lipids. DHA (22:6, n-3) is a principle component of neural membranes, accounting for 50% of the plasma membrane. **(A)** Shows the molecular structure of DHA (cmpd. 1). At carbon atom 3, the first of six double bonds is present. Despite its length, the molecule tends to make membranes thinner due to the large conformational space it occupies. Cmpd. 2 is 1-stearoyl-2-docosahexaeoyl-sn-glycero-3-phosphatidylserine, a hybrid lipid containing DHA. **(B)** Shows examples of oxidized lipid molecules. Cmpd. 1 shows an oxobutyryl derivative of phosphatidylserine. Cmpd. 2 is 4-hydroxy-2-nonenal, a degradation product of ω-6 fatty acids. Cmpd. 3 is 4-hydroxy-2-hexenal. Two additional aldehydes, malondialdehyde and acrolein, are shown as cmpds. 4 and 5, respectively. **(C)** Shows an example of a lysophospholipid generated by phospholipase A_2_ action on a PS containing a truncated fatty acid. **(D)** Shows cholesterol. **(E)** Shows 7-ketocholesterol (cmpd. 1) and 25-hydroxycholesterol (cmpd. 2), which are examples of oxysterols formed by ROS and enzymes, respectively. **(F)** Shows a cartoon illustrating key features of lipids in neural membranes. Comparatively ordered liquid domains are designated as Lo, and include lipid rafts. These regions contain cholesterol and sphingolipids. Disordered regions of the plasma membrane are termed liquid disordered (Ld). This region contains phospholipids containing DHA.

DHA is a polyunsaturated fatty acid (PUFA) constituting ~50% of a neuron's plasma membrane. DHA is esterified to phosphatidylserine (PS) or phosphatidylethanolamine (PE) in the plasma membrane's inner leaflet (Figure 1A). People obtain DHA by diet or by synthesis from dietary linolenic acid. DHA is required for normal retinal structure and vision and is neuroprotective (Uauy et al., 2001; Shimazawa et al., 2009). DHA's six double bonds cause extraordinary membrane properties. DHA-containing membranes exhibit changes in: thickness, leakiness, oxidant sensitivity, blebs, flip-flop and lateral mobility (Feller et al., 2002). Due to their thinness, DHA-containing membrane domains exhibit substantial line tension^1^ in membranes containing lipid rafts. Computational simulations show that DHA exhibits highly flexible and disordered chains in membranes, despite the rigidity of double bonds (Feller et al., 2002). DHA chains are occasionally oriented parallel to a membrane. The variety of DHA conformations is consistent with its high compressibility and low surface tension.

Plasma membranes contain two (or more) immiscible regions including liquid-disordered (Ld) and liquid-ordered (Lo) regions (Sezgin et al., 2012; Ingólfsson et al., 2014; Carquin et al., 2016). Lipid rafts, an Lo domain, were first defined by their insolubility in detergent and high cholesterol content. Lipid rafts are also enriched in saturated fatty acids and sphingolipids. DHA-containing phosphoglycerides accumulate in Ld domains because the rapid conformational changes of DHA are sterically incompatible with raft rigidity. Consequently, cholesterol is enriched in Lo regions while DHA is enriched in Ld regions of membranes (Figure 1F).

DHA is especially prone to oxidative damage. The LPP's first step is abstraction of a hydrogen atom from a lipid chain. The Gibbs free energy for removing a hydrogen atom from linoleic acid's central methylene carbon is 8 kcal/mol (Tejero et al., 2007). This is followed by addition of oxygen to form lipid peroxide (Girotti, 1998). The propagation rate constant for hydrogen extraction from oleic acid is k_p_ = 0.9 M^−1^s^−1^, whereas that of DHA is much greater at k_p_ = 321 M^−1^s^−1^ (Xu et al., 2009; Zielinski and Pratt, 2017). Moreover, high lipid and O_2_ concentrations within membranes support lipid peroxide formation. DHA-hydroperoxides promote cell death *in vivo* (Liu et al., 2008). DHA oxidation leads to phosphoglycerides containing short-chain fatty acyl derivatives with new polar functional groups, such as oxobutyryl-containing phosphoglycerides (Figure 1B). Lipid hydroperoxide fragmentation also yields small molecules such as 4-hydroxyhexenal, 4-hydroxy-2-nonenal, malondialdehyde and acrolein (Figure 1B); ~150 chemical species are formed by the LPP. 4-Hydroxyhexenal and other LPP products chemically modify retinal proteins (Tanito et al., 2005).

Oxidized phosphoglycerides change a membrane's physical properties. Membranes containing unsaturated fatty acids become thinner after 1.1 mol % of the unsaturated fatty acids have undergone peroxidation (Mason et al., 1997). The area/molecule also increases after fatty acid oxidation. The polar moieties of short chain acyl groups of oxidatively truncated phosphoglycerides (OTP) may be buried within a membrane or extend roughly parallel to a bilayer's surface with the polar moiety near the aqueous phase (Khandelia and Mouritsen, 2009). Oxidized lipids create highly disordered Ld regions that promote membrane leakiness (Runas and Malmstadt, 2015). Flip-flop across a bilayer is also enhanced, leading to scrambling of PS between the inner and outer leaflets. Oxidation of membrane lipids forms membrane pores and, ultimately, membrane dissolution (Cwiklik and Jungwirth, 2010). If unsaturated fatty acids are oxidized at both the *sn-1* and *sn-2* positions, membranes disintegrate.

Lipid head groups are also modified by oxidants. Short-chain aldehydes, such as malondialdehyde and 4-hydroxyhexenal, react with aminophospholipid head groups (Bacot et al., 2007), to become pro-inflammatory mediators. Hydroxyl radicals formed near membranes may react with phospholipid headgroups (Yusupov et al., 2017). Computational studies show that hydroxyl radicals abstract a hydrogen atom from the head group to release NC_3_${\text{H}}_{5}^{+}$, then a second hydroxyl radical forms an alcohol. Hydroxyl radicals may also attack the glycerol component of phosphogycerides to release CO_2_ and a long-chain alcohol.

To remove toxic OTPs, phospholipase A_2_ cleaves *sn-2* ester bonds to yield a free fatty acid and lysophospholipids (Figure 1C). Lysophospholipids and fatty acids behave like detergents toward membranes (Henriksen et al., 2010). Lysophospholipids reduce membrane stability and increase permeability (Arouri and Mouritsen, 2013). At 2–10 μM, lysophospholipids induce cytolytic rupture of erythrocytes. Both OTPs and lysophosphoglycerides promote apoptosis and membrane permeability at low μM concentrations *in vitro* (Arouri and Mouritsen, 2013).

By altering membrane composition, oxidants change membrane lipid distribution. Using model membranes, oxidants produced at the outer surface cause phase separations. In membranes containing POPC (1-palmitoyl-2-oleoyl-sn-glycero-3-phosphocholine), DPPC (1, 2-dipalmitoyl-sn-glycerol-3-phosphocholine) and cholesterol, the oxidation of POPC to POPC-OOH causes a lateral phase separation into two domains (Haluska et al., 2012; Itri et al., 2014). OTP exhibit the same behavior in membranes (Volinsky et al., 2012). As fatty acyl chains of OTP are shorter, membranes become thinner. For example, the height mismatch between Lo domains and Ld domains increases because truncated DHA chains cause membranes to become thinner, which increases line tension (Heberle et al., 2013; Usery et al., 2017). To minimize line tension, domains become circular in shape and, over time, coalesce into large membrane domains (García-Sáez et al., 2007; Itri et al., 2014). As damaged lipids accumulate, pores are observed in membranes (Mertins et al., 2014; Makky and Tanaka, 2015).

### Cholesterol and oxysterols

Cholesterol is a major component of plasma membranes (Figure 1D). Cholesterol promotes membrane condensation and increases mechanical stability. It packs with sphingolipids to form membrane domains known as rafts, which regulate cell functions (Lingwood and Simons, 2010). Cholesterol levels and thereby membrane rafts, are tightly regulated by cells. To achieve tight regulation, cells use multiple means of cholesterol management. Cells obtain cholesterol by endogenous synthesis and import cholesterol from the bloodstream via low density lipoproteins. Cells dispose of excess or damaged cholesterol by modifying it into a more hydrophilic form that leaves cells by diffusion and by transporting it from cells via high density lipoproteins.

Cholesterol is oxidized by enzymes and ROS to yield oxysterols (Figure 1E). CYP27A1 is a cholesterol 27-hydroxylase whose product, 27-hydroxycholesterol, regulates cholesterol synthesis. CYP46A1 converts cholesterol to 24S-hydroxycholesterol. Singlet oxygen reacts with the double bond of cholesterol's ring to yield 5- and 7-hydroperoxide derivatives. Chemical rearrangements lead to 7-hydroxycholesterol and 7-ketocholesterol. Hydroxyl radicals react with cholesterol (k_p_ = 11 M^−1^s^−1^) to yield cholesterol 7-hydroperoxide and 5,6-epoxycholesterol. Cholesterol 7-hydroperoxide can yield 7-hydroxycholesterol that, in turn, can be oxidized to 7-ketocholesterol. 5,6-Epoxycholesterol may further react to form 5, 6-dihydroxycholesterol.

Cells can convert excess cholesterol to side-chain oxysterols. As hydroxycholesterols are hydrophilic, they passively cross tissue barriers. This pathway participates in excess cholesterol clearance from the brain. As cholesterol turnover is much higher in the retina than the brain (Rodríguez and Larrayoz, 2010), this pathway is unlikely to be important in retina. Side-chain oxysterols are ligands for liver X receptors (LXR). LXRs increase the expression of genes participating in cholesterol export from cells, such as ABCA1 (ATP binding cassette transporter A1). Side-chain sterols also suppress the transcriptional factor sterol response element binding protein (SREBP). Activated SREBP up-regulates genes participating in cholesterol synthesis (HMGCoA reductase) and uptake (LDL receptor). Thus, oxysterols reduce the production and internalization of cholesterol. Oxysterols promote ubiquitination of HMGCoA reductase, which targets it for destruction by proteasomes. Cholesterol and oxysterols also regulate acyl-CoA cholesterol acyl transferase (ACAT), which couples cholesterol to fatty acids to form cholesteryl esters for storage as cytoplasmic lipid droplets.

Oxysterols have biological effects at a wide variety of concentrations. They bind with high affinity (K_d_~5 nM) to oxysterol binding proteins (OSBP) and OSBP-related proteins (ORP) (Raychaudhuri and Prinz, 2010). OSBPs influence lipid metabolism, signaling, membrane contact sites, cytoskeletal regulation, and lipid transfer. For example, OSBP1 is expressed by Müller cells (Moreira et al., 2001). It binds cholesterol originating from lysosomes, and facilitates its transport to the nucleus where it downregulates LDL receptors, HMG-CoA reductase and HMG synthetase. Oxysterols induce apoptosis; for example, 7β-hydrocholesterol, 7-ketocholesterol and cholesterol-5β,6β-epoxide induce apoptosis at 20–30 μM (e.g., O'Callaghan et al., 2001; Larrayoz et al., 2010). Although the oxysterol-cholesterol ratio is 1:1000 in normal tissues, the oxysterol-cholesterol ratio climbs to 1:5 during certain pathological conditions (Tint et al., 1995; Javitt, 2008; Brown and Jessup, 2009; Olkkonen et al., 2012; Kulig et al., 2016). Therefore, it seems likely that oxysterols have physiological effects during pathological conditions.

Although cholesterol packs tightly with other lipids to form rafts, oxysterols do just the opposite—they promote membrane expansion and leakiness. Not surprisingly, 7β-hydroxycholesterol causes a 32% reduction in the lysis tension of giant membrane vesicles (Kim and Frangos, 2008). 27-hydroxycholesterol and 25-hydroxycholesterol have polar groups at both ends of the molecule, suggesting that they interact with membranes in unusual ways (Hilsch et al., 2017). Biophysical studies show an increase in fatty acyl chain order as they pack around cholesterol molecules. In contrast, 25-hydroxycholesterol increases membrane order at the lipid head group, but decreases order at fatty acyl tails. 25-Hydroxycholesterol has two membrane conformations: one similar to cholesterol, with a hydroxyl group buried in the membrane, and a second conformation roughly parallel to the membrane's surface. Insertion of 25-hydroxycholesterol into an RBC membrane causes its outer leaflet to expand relative to the inner leaflet, resulting in echinocyte transformations (Hsu et al., 1980). 25-Hydroxycholesterol promotes morphological effects in nucleated cells including the disappearance of microvilli and blebbing (Lin and Chen, 1979), which may be explained by lipid raft disruption and/or OSBP action on the cytoskeleton.

Lipid raft functions are often studied using methyl β-cyclodextrin to extract cholesterol from membranes. In a more elegant approach, membrane cholesterol is replaced with oxysterols. 7-Hydroxycholesterol and 7-ketocholesterol are slightly tilted in membranes due to the functional group at the ring's 7 position. In untreated cells, EGF signaling is intact, but cholesterol depletion abrogates signaling. If cholesterol is returned to the cells, signaling returns; interestingly, 7-ketocholesterol only partially restores signaling (Massey, 2006). Hence, small changes in sterol structure and packing effect signaling.

In addition to interfering with cell signaling, oxysterols also promote cytotoxicity by influencing enzyme activities. Cells protect themselves from high cholesterol levels by esterification of cholesterol to fatty acids, then sequestering the ester in cytoplasmic droplets. Cytotoxic oxysterols, such as 7-ketocholesterol, are poor substrates for the acyl transferase, thereby interfering with cholesterol management. Furthermore, 7-ketocholesterol is a poor substrate for the ATP-binding cassette transporter A1 (ABCA1) and ABCG1, and, in fact, interferes with this enzyme. 7-Ketocholesterol is also believed to activate an ER-associated NADPH oxidase by enhancing ER membrane order to produce ROS. Thus, the generation of oxysterols is enhanced by oxysterols themselves while strategies to manage oxysterols are blocked.

## A physical theory of glaucoma

During POAG the IOP applies a compressive stress on ocular tissues. I propose a chemical-mechanical coupling model of glaucoma wherein the IOP's constant perpendicular stress on a plasma membrane (*S*_IOP_) couples with the reduced surface strength of glaucomatous RCG membranes (*S*_memb_ – Δ*S*_LPP_) to nucleate membrane pores (**Figure 3C**). The accumulation of oxidatively damaged lipids (see above) reduces membrane compressive strength (and tensile strength)^2^. When the IOP exceeds the RGC membrane's compressive strength (Equation 1), pores form.

(1) $$\begin{array}{l} {\text{S}}_{\text{IOP}}>{\text{S}}_{\text{memb}}-\Delta {\text{S}}_{\text{LPP}} \end{array}$$

A constant perpendicular stress of ~12 kPa is sufficient to rupture cell membranes (Hategan et al., 2003; Peeters et al., 2005; Gonzalez-Rodriguez et al., 2016). However, cellular measurements of compressive and tensile strength include a significant contribution from cytoskeletal structures (Lulevich et al., 2006, 2010; Sánchez et al., 2008), which act to resist cell damage and confounds analysis of puncture formation in cell membranes. For example, deformation studies of intact neurons observed two Young's moduli of 0.5 kPa, corresponding to membrane deformation, and 6.9 kPa corresponding to membrane + cytoskeletal deformation (Sánchez et al., 2008). The cytoskeleton's role in the mechanical properties of cell surfaces has been widely studied (e.g., Lulevich et al., 2010; Gefen and Weihs, 2016). In this analysis, the stability of the cytoskeleton is not important, we are only interested in the stability of the bilayer membrane toward pore formation. For pedagogical purposes, we conservatively estimate a compressive strength of 7 kPa for neuronal membrane bilayers.

A second reason for the overestimation of cell membrane compressive strength is that RGC membranes contain high PUFA levels. Monounsaturated and PUFA reduce a membrane's tensile strength (Needham and Nunn, 1990; Zhelev, 1998) and significant changes in compressive strength are anticipated. This is confirmed by the very low Young's modulus of neuronal cells (Sánchez et al., 2008). An actual neuronal membrane bilayer may have a compression strength of ~5 kPa.

During glaucoma, extensive oxidative damage occurs (see above), especially in membrane lipids. These lipids do not support bilayer formation, and can therefore be very damaging to cells. For example, lysophospholipids at 30 mol% dramatically reduce the tensile strength of membranes and at 50 mol% lysophospholipid, tensile strength disappears (Zhelev, 1998). OTP, oxysterols, aldehydes, lipid peroxides and hydroperoxides also reduce membrane strength. Pore formation is a localized phenomenon. Due to their small size, damaged lipids such as fatty acids, lysophosphoglycerides, and OTP will accumulate in lipid domains enriched in these molecules due to line tension. These regions, which need not be large, will be the first to fail during glaucoma. The compressive strength of these regions is reduced; ~2–3 kPa is a reasonable estimate. This estimate is smaller than the IOP of 4 kPa (30 mmHg) in glaucoma patients. Thus, our hypothesis is consistent with the physical and the chemical properties of the ocular environment.

### Membrane pores

Direct microscopic observations show that erythrocytes undergo catastrophic ruptures during leukocyte-mediated cytolysis, which is promoted by ROS formation (Francis et al., 1988). In this case, it was possible to estimate the pore's radius (~30 nm). On the other hand, leukocyte-mediated attacks on tumor cells lead to numerous transient membrane pores (Kindzelskii and Petty, 1999). These differences may be accounted for by cellular survival mechanisms and/or the membrane's physical properties. Lymphocytes promote membrane pore growth in tumor cells by increasing the local membrane tension, not the cortical tension of the cytoskeleton (Basu et al., 2016). Thus, weakening a membrane's bilayer structure and/or increasing its surface tension promote membrane pores leading to cell death.

A pore's ability to grow into a large rupture is determined by the surface tension, which acts to open a pore, and line tension about a pore's perimeter, which closes the pore (Sandre et al., 1999). The energy, E, of a pore of radius r is:

(2) $$\begin{array}{l} E_{\text{r}}=2\pi \text{r}\gamma -\pi {\text{r}}^{2}\sigma \end{array}$$

where γ = line tension and σ = surface tension. Pores close when r < 2γ/σ, but expand when r>2γ/σ. Using σ = 0.04 mN/m for nucleated mammalian cells (Gauthier et al., 2012) and γ = 5 pN, which has been estimated for membranes containing PUFAs (Rosetti et al., 2017), a value of 2γ/σ = 250 nm is obtained. It seems unlikely that nucleation sites for pore growth of this size will occur, suggesting that multiple transient pores will be observed. Membrane ruptures grow rapidly when a pore's line tension is small in comparison to surface tension. On the other hand, pores close when line tension is large in comparison to surface tension.

We now see that high surface tension is dangerous for cells because it supports a run-away surface rupture (Equation 2). But very low surface tension is also dangerous for cells: as surface tension falls to zero, membrane tensile strength disappears, thus leading to the same outcome. If chemical conditions reduce surface tension in the presence of an external pressure, as illustrated in Equation (1), membrane pores will form, which, if not managed, will lead to cell death. Thus, mammalian cells must maintain their surface tension within a range to support viability.

### Damage and repair

When a membrane's line tension dominates, small pores will spontaneously close. Large ruptures formed by pore expansion, large mechanical stress, or physical intrusion into a cell, may not spontaneously close. For structures lacking endomembranes, such as liposomes and erythrocytes, the kinetics of label release provide information regarding pore size. The kinetics of label release from liposomes and erythrocytes are about 10–100 s and 1–5 s, respectively (Lewis and McConnell, 1978; Francis et al., 1988), which correspond to pores of roughly 30–100 nm in size. The kinetics of membrane rupture for cells containing endomembrane structures is more complex (McNeil et al., 2003). These cytolytic ruptures are managed by active repair mechanisms (McNeil and Steinhardt, 1997; Tang and Marshall, 2017). This mechanism relies upon the rapid calcium-dependent exocytosis of vesicles near a rupture, which increases surface area, reduces surface tension, and allows the hole to close. For example, using a 0.5 μm microneedle to impale 3T3 cells, Shen et al. (2005) have observed that wounds close in about 30 s. As an endomembrane system is present in 3T3 cells, the puncture closes faster than expected in the absence of endomembranes. After more substantial damage to an axon, membrane vesicles are transported to the breach where they aggregate to plug a rupture—such as transection of an axon (Krause et al., 1994). Thus, membrane damage could spontaneously heal or be repaired by active mechanisms that rely upon endomembranes and vesicular trafficking.

In POAG, the presence of heightened IOP and oxidized lipids lead to pore formation (Equation 1). Pore formation could be stimulated at normal IOPs with greater levels of oxidative damage. Although the pore may close, the lipids remain and could reform another pore within the same membrane at a later time. Lipid recycling in this way is expected to continually drive PS into the outer leaflet of the plasma membrane. Moreover, membrane ruptures stimulate NADPH oxidase-dependent ROS production (Arbault et al., 1997), which accelerates pore formation. These ROS will further stimulate the LPP and damage membranes. We will now consider the genetic factors that may permit this type of membrane damage.

## Glaucoma-susceptibility gene polymorphisms and their role in membrane stability and disease

**Figure 2:**
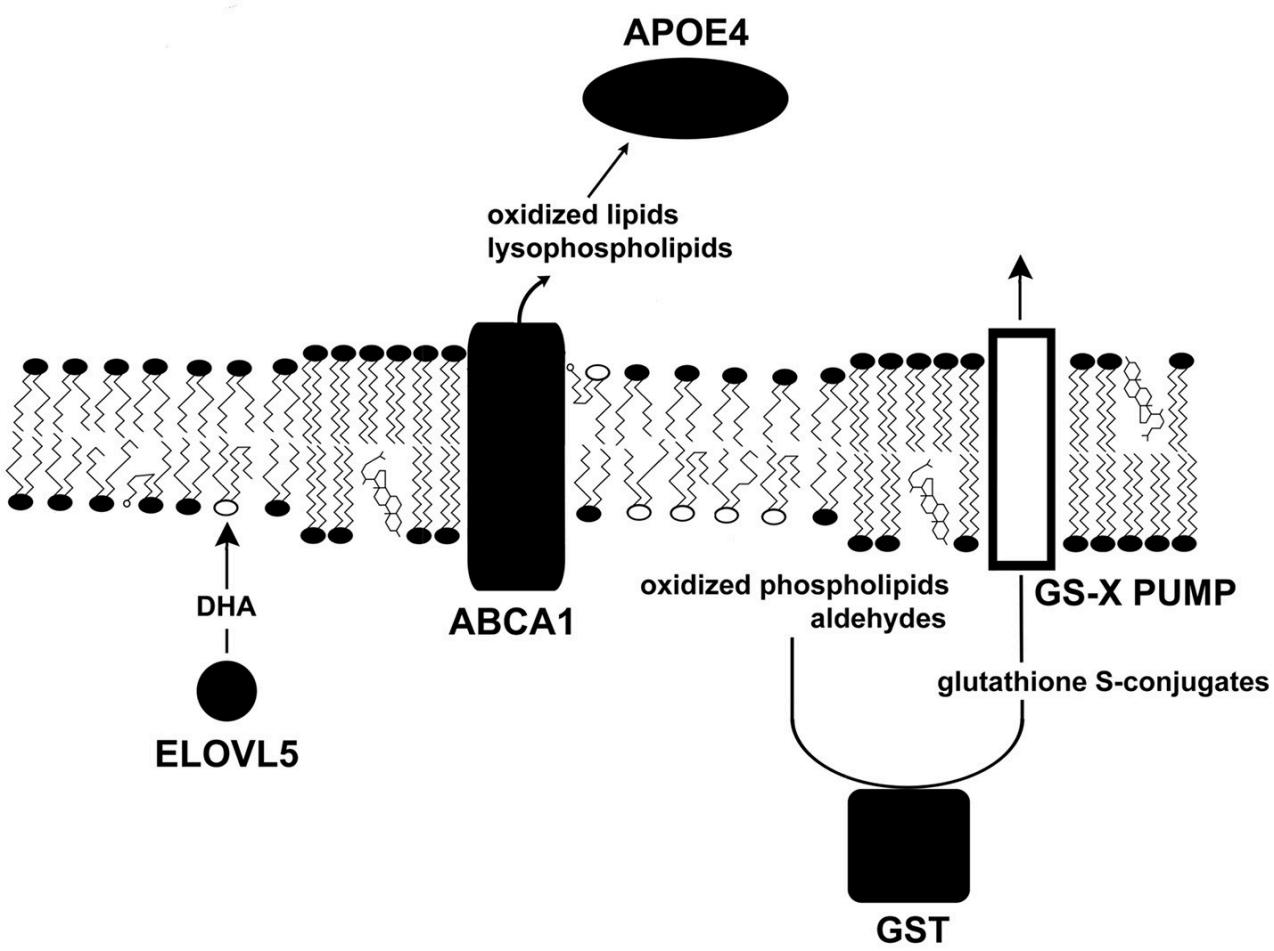
Several glaucoma susceptibility genes influence surface tension. This diagram illustrates the roles of several proteins that confer susceptibility to glaucoma (solid shapes). Other glaucoma susceptibility genes are upstream, where they heighten IOP, while others are downstream affecting apoptosis. ELVOL5 catalyzes the first step in the synthesis of DHA, which acts as a target for oxidants. Phospholipase activity removes damaged fatty acids from phospholipids. ABCA1, an ATP-driven pump, removes oxidized lipids and lysophospholipids from cells. APOE4 transports damaged molecules to the liver for recycling. GST couples harmful lipid species to glutathione for cell export.

Genetic factors play a key role in POAG. Genetic linkage analyses of families are performed to identify chromosomal regions associated with a disease phenotype. Linkage analysis led to the discovery of the myocilin gene (*MYOC*), which causes glaucoma. This monogenic form of glaucoma will be discussed in this section, as it fits well with this paper's theme. However, it is difficult to perform linkage analyses because pedigrees are difficult to obtain for POAG patients because of their age. Genome-wide association studies (GWAS) are another method to extract genetic information about a disease. Several million single nucleotide polymorphisms (SNP) may be analyzed for each patient sample to locate disease-associated polymorphisms. These polymorphisms may be found in exons or introns within a gene or other nearby regions of the chromosome. Polymorphisms could affect the phenotype at many levels including the mature gene product or its intracellular trafficking as well as it expression and regulation. This approach has been very productive. Genes correlating with glaucoma risk may involve: ocular development, intraocular pressure, RGC membranes, ROS production and management, and mechanisms controlling apoptosis. For the present discussion, glaucoma-susceptibility genes expected to influence membrane stability will be presented (Figure 2).

### ELOVL5

*ELOVL5* gene polymorphisms have been associated with susceptibility to high and normal tension glaucoma (Meguro et al., 2010; Mabuchi et al., 2011, 2015). The endoplasmic reticulum enzyme ELOVL5 (elongation of very long chain fatty acids 5) catalyzes the first step of the fatty acid elongation cycle, which contributes to the synthesis of long chain fatty acids including DHA. *ELOVL5* knock-out mice accumulate ELOVL5 substrates whereas its products, arachidonic acid and DHA, are reduced (Moon et al., 2009). It seems likely that reduced levels of DHA lead to increases in oxidative damage in neurons.

### ABCA1

GWAS show that an ABCA1 polymorphism predisposes individuals to glaucoma (Chen et al., 2014; Gharahkhani et al., 2014; Hysi et al., 2014). *ABCA1* encodes an ABC transporter with 12 transmembrane α-helices. ABCA1, the first member of the A family) is expressed in all retinal layers and the optic nerve. Using ATP as an energy source, ABCA1 promotes the removal of cholesterol, oxidized lipids, and lysophospholipids from cells (Tam et al., 2006). ABCA1 also acts as a receptor for apoproteins to enhance delivery of lipids to apoproteins. ABCA1 interacts with lipid-poor APOA-I, APOA-II, and APOE to promote lipid export. The removal of OTP, oxysterols, and lysophospholipids is essential to stabilize membranes. Damaged lipids are then transported to the liver via lipoproteins. ABCA1 expression is upregulated by the peroxisome proliferator-activated receptor γ (Chinetti et al., 2001). ABCA1 activity is influenced by membrane domains and caveolae (see below). The regulation of ABCA1's functional ability is very complex, and likely to be broadly important in many diseases and tissues.

### ApoE4

APOE is a plasma protein produced by the liver and peripheral tissue. The *APOE* gene contains 4 exons. This gene is polymorphic with three common *APOE* isoforms encoded by different alleles (ε2, ε3, and ε4). APOE is a protein of 299 amino acids containing amphipathic helices. It is made up of amino terminal and carboxyl terminal domains linked together by a hinge region. The three polymorphisms correspond to sequence variations in the N-terminal domain positions 112 and 158 wherein cysteine residues or arginine residues may be found (Morrow et al., 2000). These isoforms vary in structure and function and contribute to susceptibility to several degenerative neural disorders. APOE produced in the liver is primarily found as a component of VLDL, which transports triglycerides, phospholipids, cholesterol, and cholesteryl esters to tissues. Reverse lipid transport also takes place wherein excess cholesterol in tissues is transported to the liver via HDL. In tissues, APOE2 and APOE3 preferentially bind to HDL whereas APOE4 binds to VLDL.

APOE4 has been associated with an elevated risk of developing POAG (Al-Dabbagh et al., 2009; Liao et al., 2014; Wang et al., 2014). Although the mechanism accounting for POAG susceptibility for *APOE* ε4 carriers is unknown, it is known that APOE4 appears to carry lipids less efficiently than APOE3 (Gong et al., 2002). *In vitro*, APOE3 is 2.5–3.9-fold better at promoting lipid efflux from astrocytes than APOE4 (Minagawa et al., 2009). It is possible that lipid trafficking plays a role in disease. In patients given supplemental DHA, the *APOE4* allele was associated with less DHA in the cerebrospinal fluid than other alleles (Yassine et al., 2016). Thus, APOE4-bearing individuals may have limitations in lipid management.

Apolipoproteins cooperate with ABCA1 and other proteins to manage lipids. HDL is a major carrier of lipid hydroperoxides (Bowry et al., 1992), but they are more than just vehicles to support lipid trafficking. HDLs catalytically reduce cholesteryl ester hydroperoxides and phosphatidylcholine hydroperoxides (Garner et al., 1998). The APOAI and APOAII catalyze a two electron reduction of lipid hydroperoxides to their corresponding hydroxides. This step is particularly important because lipid peroxides damage membranes and promote LPP activity.

### Glutathione S-transferases

**Figure 3:**
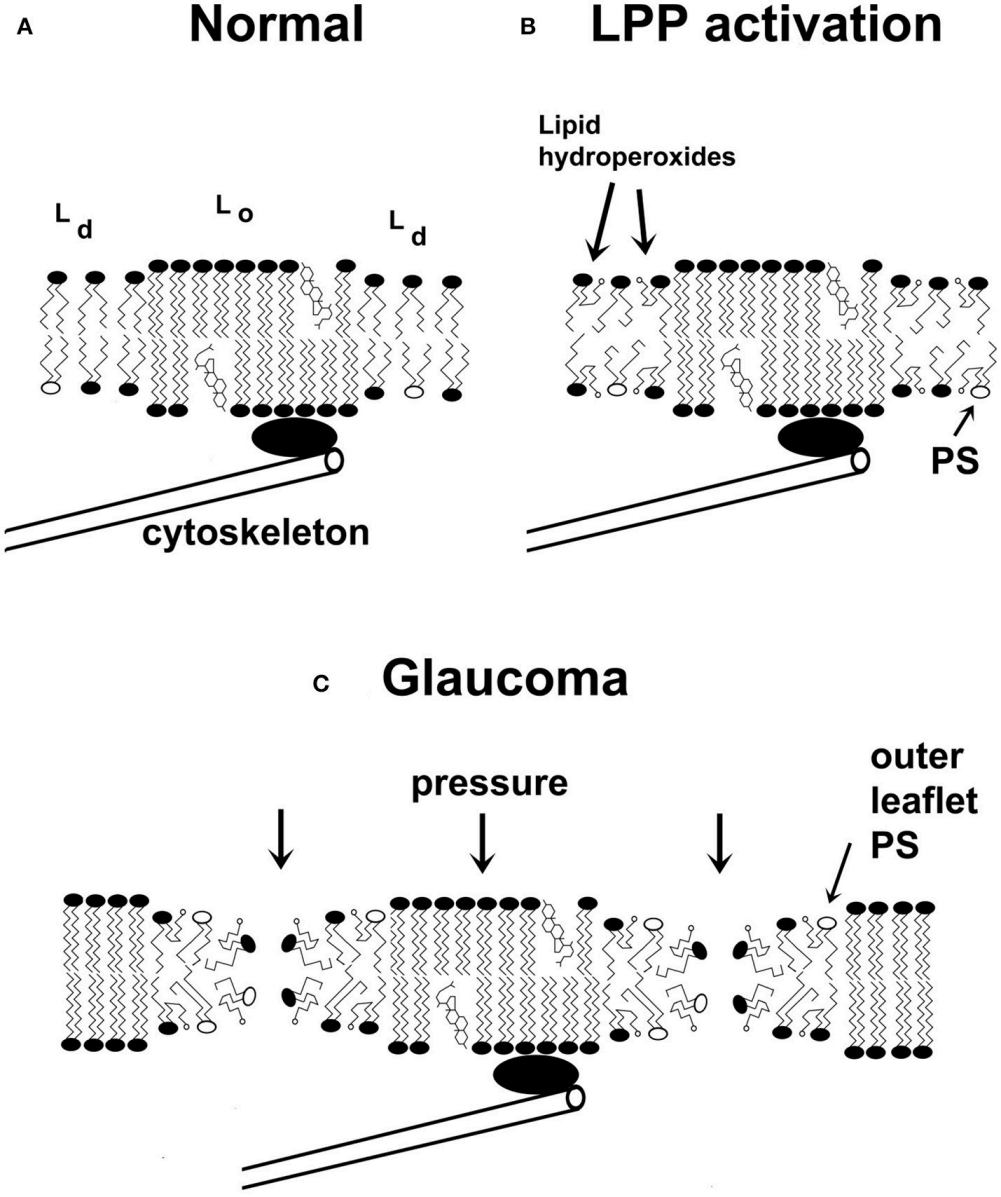
A schematic two-dimensional drawing of cell membranes. **(A)** Cell membranes are characterized by liquid-disordered (Ld) and liquid-ordered (Lo) regions, the latter group includes raft domains. The interface between the Lo and Ld regions is characterized by line tension, which arises from the mismatch in membrane thicknesses of the Lo and Ld domains. **(B)** Under specific conditions, lipid peroxidation products (lipid hydroperoxides are shown) accumulate in the membrane's Ld phase. This further reduces the thickness of the fluid domains and increases the line tension. Due to the insertion of carboxylic acid groups into the acyl chain of the lipids, the conformation of the lipids changes, which destabilizes the membrane. **(C)** Membranes weakened by activation of the lipid peroxidation pathway are more susceptible to catastrophic breakdown in the presence of a heightened IOP, leading to the formation of multiple transient or permanent membrane leaks. One consequence of membrane integrity changes is that PS will accumulate at the outer surface of the membrane, thus promoting the recognition of the cell as apoptotic. Non-bilayer membrane changes could also lead to disrupted action potentials. Carboxylic acids of lipid hydroperoxides are indicated by small open circles. Phosphatidylserine (PS) molecules are shown as phospholipids with open ovals as headgroups. (Some features are exaggerated for pedagogical purposes).

Glutathione S-transferases (GSTs) are cytosolic, mitochondrial, and ER enzymes that couple reduced glutathione to xenobiotics, oxidized DNA bases, peroxidized lipids and their products, such as aldehydes. Consequently, these dangerous molecules become more hydrophilic, and more amenable to transport out of a cell. Five classes of GSTs have been identified in humans (α, μ, π, θ, ζ). GSTM1 null mutations, those with a complete loss of function, have been associated with a predisposition to develop POAG (Juronen et al., 2000; Unal et al., 2007; Abu-Amero et al., 2008, 2009; Huang et al., 2013; Lu et al., 2013; Gao et al., 2018) and other optic neuropathies (Abu-Amero et al., 2009). Studies of the potential role of GSTT1 in POAG have produced conflicting results. Consequently, null mutants of GSTM1 exhibiting no GST activity will be less able to tag at least a sub-population of toxic electrophiles for removal via membrane pumps (Ishikawa, 1992; Figure 3), which allows membrane-destabilizing molecules to accumulate.

### Cytochromes P450

Polymorphisms *CYP46A1* in intron 2 predispose individuals to POAG (Fourgeux et al., 2009). Presently, it is difficult to predict how this intron affects phenotype. CYP46A1 converts cholesterol to 24S-hydroxycholesterol in neurons, including RGCs. Fourgeux et al. (2009) report that the polymorphism is not associated with changes in serum 24S-cholesterol levels. However, the basal levels of this metabolite in serum are far too high to detect any small change that might be due to ocular production. This polymorphism's action could be explained by an absence of protective effect(s). Ishikawa et al. (2016) have shown that 24S-hydroxycholesterol protects retinal cells from injury at high hydrostatic pressure. Elevated hydrostatic pressure increases *CYP46A1* gene expression and that of its product. Moreover, it might be expected that *CYP46A1* polymorphisms leading to local reductions in 24S-hydroxycholesterol concentrations are also less able to stimulate LXR receptors, thereby diminishing ABCA1 up-regulation and damaged lipid export. Finally, the up-regulation noted by Ishikawa et al. (2016) will introduce 24S-hydroxycholesterol into the plasma membrane, which will expand the bilayer and promote compressibility. Thus, heightened 24S-hydroxycholesterol could alter the physical properties of membranes (i.e., Young's modulus) to protect a cell from ruptures at high IOP episodes; this might not be possible with dysregulation of expression, which might be observed for intronic mutations.

### Myocilin

Myocilin (*MYOC*) gene mutations are linked to monogenic POAG, especially the juvenile form (Kwon et al., 2009). Myocilin is expressed in the trabecular meshwork, the optic nerve head, and other tissues (Clark et al., 2001). Gene sequencing has revealed a signal sequence in myocilin's primary structure, which has led to the suggestion that it is a secretory protein. However, this interpretation may be wrong because signal sequences are also used to deliver proteins to membranes and the cytosol. *MYOC* contains three exons: an N-terminal myosin-like region, a central coiled-coil region, and C-terminal olfactomedin-like region. Myocilin binds to the myosin motor complex (Wentz-Hunter et al., 2002), suggesting a role in intracellular trafficking. Mutant myocilin has been linked to aberrant microfilament assembly in RGCs (Ying et al., 2012). Myocilin is associated with intracellular vesicles and exosomes (Hardy et al., 2005). The central coiled coil region associates with membranes (Dismuke et al., 2012). A portion of the coiled coil region is homologous to Q-SNAREs (soluble N-ethylmaleimide-sensitive factor attachment protein receptors) (Dismuke et al., 2012), which participate in vesicle docking and fusion. Myocilin's third region, the olfactomedin domain, has been linked to most disease-causing variants. It has a unique Ca^2+^-binding domain that has been linked to several *MYOC* mutations (Donegan et al., 2012). This Ca^2+^-binding site has a K_d_ ~10^−6^M (Donegan et al., 2012). This K_d_ is too high to meaningfully react to physiologically-relevant changes in intracellular Ca^2+^, but too low to respond to changes in extracellular Ca^2+^ concentrations. However, the K_d_ seems to be appropriate to detect large increases in intracellular Ca^2+^ that accompany membrane lesions. Hence, I propose that myocilin participates in vesicle delivery to damaged membrane regions, thereby promoting membrane fusion, reducing surface tension, and repairing membrane damage.

Studies of mice with targeted null *MYOC* mutations were unable to detect a disease phenotype, suggesting that its effect is mediated by a gain-of-function (Kim et al., 2001). However, there is no affirmative evidence for a gain-of-function, only a lack of evidence for a phenotype. It is possible that a *MYOC* phenotype exists, but only when it is called upon to function. As ~100 *MYOC* mutations have been reported (www.myocilin.com), it seems unlikely that they are all rare gain-of-function mutations; moreover, there is no phenotype associated with the proposed gain-of-function. I suggest that myocilin's mutations lead to the loss of biological function.

Although small pores may close spontaneously or with the assistance of endomembranes, large ruptures may require more extensive responses. Such membrane lesions could be plugged by aggregated vesicles. The appearance of myocilin-bearing exosomes in the aqueous humor (Perkumas et al., 2007) is possibly due to an accumulation of old plugs previously used to protect damaged ocular membranes. This interpretation is consistent with the fact that extracellular myocilin is not observed in patients with sight-threatening myocilin mutations (Jacobson et al., 2001). This concept is consistent with a disturbance in the secretory pathway suggested by Jacobson et al. (2001). Moreover, exocytosis relies upon calcium entry into cells, which is mediated by pores, ionophores or steroids. Ionophores and steroids have been shown to promote myocilin-vesicle release in trabecular meshwork cells *in vitro* (Hoffman et al., 2009). This suggests that myocilin may play a role in vesicle translocation for RGC (or trabecular cell) membrane repair; significant disruption of repair mechanisms may contribute to myocilin's association with juvenile POAG.

### Caveolin

Polymorphisms located in the intergenic region between *CAV1* and *CAV2* genes have been identified as POAG risk factors (Thorleifsson et al., 2010), although it is uncertain how these polymorphisms affect caveolin function. Caveolins are associated with specialized membrane invaginations known as caveolae. During ABCA1 up-regulation, caveolins dissociate from caveolae, then remain uniformly distributed on membranes (Landry et al., 2006). This causes caveolae membranes to intermix with the bulk plasma membrane. This intermixing or “unpacking” of membranes may facilitate the removal of cholesterol and other lipids by ABCA1 (Landry et al., 2006). A role of caveolin in membrane repair is consistent with studies showing that cells disassemble caveolae during mechanical stress (Sinha et al., 2011) and that caveolea disassembly protects endothelial cells from rupture (Cheng et al., 2015). Hence, caveolin may contribute to damaged lipid removal, reduced surface tension, and increased surface area during mechanical stress.

It is possible that additional genes predisposing individuals to glaucoma may participate in membrane repair (Kumar et al., 2016; Liu and Allingham, 2017; Wiggs and Pasquale, 2017). Optineurin (OPTN), which has been principally associated with normal tension glaucoma, is a possible participant in membrane repair because it has been linked to membrane trafficking (Kachaner et al., 2012). TMCO1 (transmembrane coiled-coil domains 1) is another protein that may fit with membrane repair. TMCO1 is an endoplasmic reticulum calcium signaling protein (Wang et al., 2016) that has been associated with POAG (Liu and Allingham, 2017). As calcium signaling has been linked to membrane repair, TMCO1 may participate in mobilizing the vesicle-mediated repair mechanisms. Actin filament associated protein 1 (AFAP1) variants have been associated with POAG (Gharahkhani et al., 2014; Bailey et al., 2016). AFAP1 is a src-binding partner that is believed to contribute to microfilament integrity and signaling. It is possible that this protein also participates in the trafficking of vesicles to sites of membrane damage. As the protein products associated with a predisposition to glaucoma become better understood, additional mechanistic details should be revealed.

## Proposed mechanisms of disease

**Figure 4:**
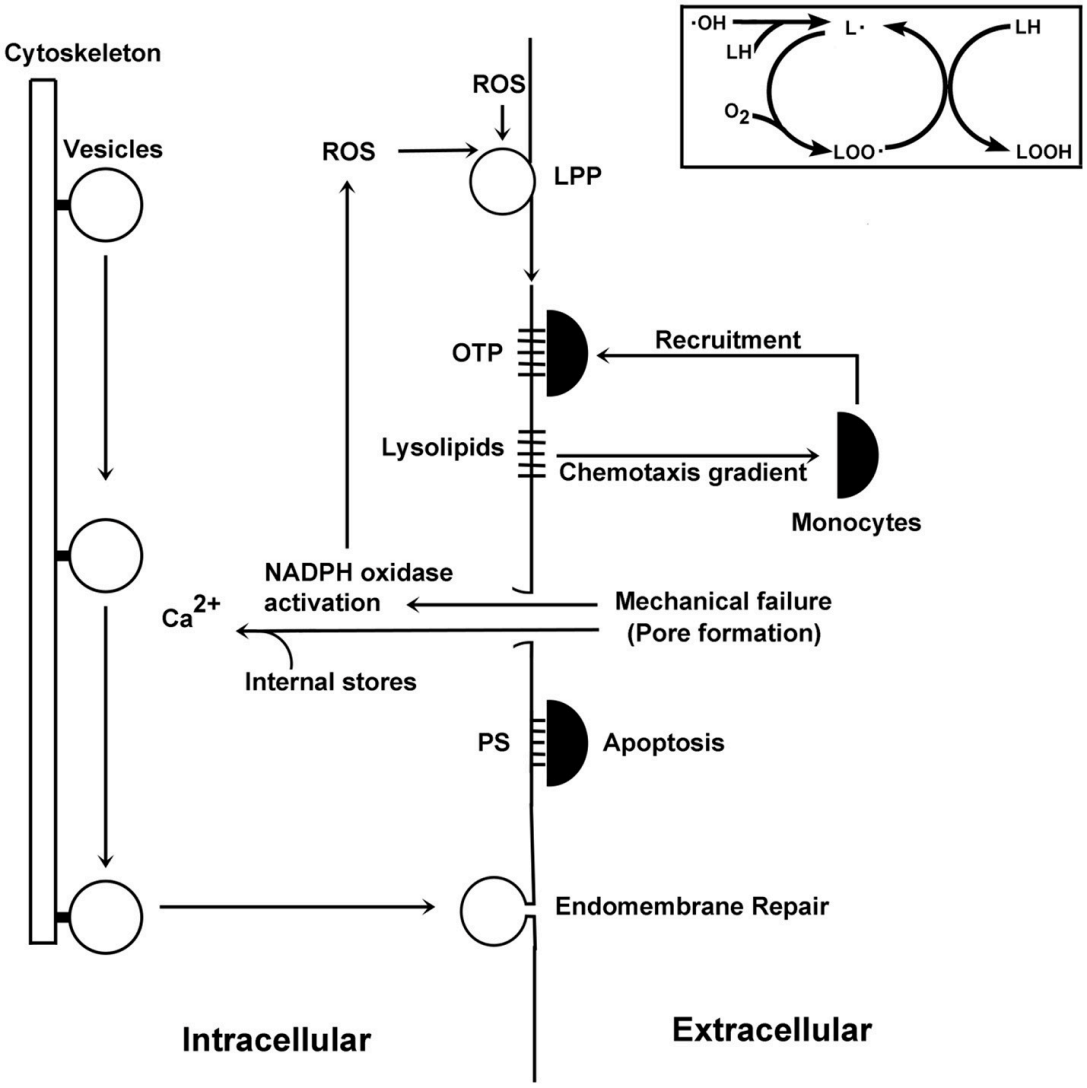
Schematic illustration of events surrounding pore formation. ROS promote the formation of lipid radicals (see insert on the upper right hand side). These molecules react with oxygen to form lipid peroxides, which can then react with other lipids to form lipid hydroperoxides and reform another lipid radical. This autocatalytic cycle can form many lipid hydroperoxides for each radical entering the pathway. Downstream products include lysolipids, which are chemotactic factors for monocytes, and OTP, which interact with monocytes. As damaged lipids accumulate, their physical properties lead to mechanical failures of the plasma membrane. Membrane damage activates the NADPH oxidase, which synthesizes ROS thereby creating more damaged lipid species and so on. These membrane pores also allow for the entry of calcium from the extracellular environment to enter the cell, which stimulate calcium-dependent vesicle-mediated endomembrane repair processes. The membrane discontinuity created by membrane pores allows PS to traffic to the external leaflet of the plasma membrane, where it can be recognized by monocytes to initiate apoptotic processes.

POAG takes a slow course, with symptoms appearing suddenly after significant damage to RGCs. This observation is consistent with Equation (1): either the membrane is stable or it is not. The symptoms are also consistent with the underlying non-linear chemical feedback systems in glaucoma: the LPP and pore-dependent activation of the NADPH oxidase. These two pathways are linked in series—each is driven by products of the other pathway (Figure 4). Once activated, it is easy to see how the system could be driven away from homeostasis. The slow course of disease may be due to the time required for the accumulation of damaged lipids and pore nucleation in membranes. The lipid disposal machinery plays a role in the maintenance of membrane stability, and polymorphisms that negatively impact this process should promote disease. As many genes may contribute to processes affecting membrane integrity, POAG is seen as a polygenic disease.

Due to their complexity, polygenic disease mechanisms have been elusive. Several glaucoma-susceptibility gene polymorphisms are associated with lipid management and membrane repair. ELOVL5 participates in DHA synthesis. DHA and cholesterol are important because they quickly react with oxidants, but in doing so they initiate the LPP and the formation of dangerous products. OTP, lysophospholipds, fatty acids, and other molecules reduce membrane strength, which promotes membrane leakiness and pore formation. Due to their physical properties, damaged lipids should form separate domains. Indeed, lipid metabolite-enriched membrane domains are present in cell (Madey et al., 2001) and artificial membranes (Haluska et al., 2012; Volinsky et al., 2012; Itri et al., 2014; Runas and Malmstadt, 2015). The line tension formed by the hydrophobic mismatch of oxidatively damaged lipids is expected to promote the formation of modified Ld domains (Volinsky et al., 2012; Itri et al., 2014) enriched in damaged lipids. As lipid waste products must be removed to maintain membrane stability, polymorphisms reducing damaged lipid disposal will favor membrane damage, which becomes evident later in life as glaucoma.

An important element in waste management is ABCA1, which cooperates with apolipoproteins to remove damaged lipids from cells. APOE4 is less effective than other APOE variants in mediating trafficking of ingested DHA. GSTs are also engaged in lipid waste removal. GSTs conjugate peroxidized lipids, aldehydes, and related molecules to glutathione thus enabling these molecules to be transported out of cells. Polymorphisms of *CYP46A1* reducing 24S-hydroxycholesterol levels diminish *ABCA1* up-regulation and therefore cells would be less able to remove oxidized lipids and their products. When caveolins dissociate from caveolae, a cell's surface area will increase. Under normal conditions, this activity would decrease surface tension and potentiate damaged lipid removal via ABCA1. Certain oxysterols at low concentrations are autocrine mediators that regulate cells via OSBPs. At somewhat higher concentrations, oxysterols influence the properties of lipid rafts to garble normal signaling events. Still higher concentrations are cytotoxic, possibly due to their conformational properties in membranes (Wielkoszynski et al., 2006). As oxidants degrade a membrane's strength, the combined effect of weakened membranes and heightened IOP promote pore formation. Pore formation is evidenced by the leakage of cytoplasmic lactate dehydrogenase —a marker of membrane rupture—into the aqueous humor of glaucoma patients (Jovanovic et al., 2010). Transient pore formation is consistent with an absence of early structural changes in POAG (Anderson, 1972). Oxidant-mediated pore formation has been observed in other cell systems (Francis et al., 1988; Kindzelskii and Petty, 1999). Pore formation allows phosphatidylserine to redistribute between the bilayer leaflets (Figure 3C), which promotes apoptosis (Asano et al., 2004). Moreover, OTP at low μM levels cause mitochondrial depolarization, which also contributes to apoptosis (Chen et al., 2009).

Low-grade inflammation has long been suspected in glaucoma (Vohra et al., 2013). Lipid hydroperoxides released from cells are chemotactic factors for mononuclear leukocytes (Peter et al., 2012) and can propagate chemical damage to neighboring cells. Lysophospholipids are also chemotactic for monocytes, thus suggesting potential pro-inflammatory signaling (Quinn et al., 1988; Duong et al., 2004; Peter et al., 2012). The polar moieties of oxidatively truncated fatty acids of damaged phosphoglycerides project into the aqueous space. This moiety is recognized by CD36, a pattern-recognition receptor of monocytes (Greenberg et al., 2008). As monocytes congregate at RGCs, they recognize surface phosphatidylserine to trigger apoptosis.

## Potential role of membrane stability in additional diseases

ROS and oxidative tissue damage are observed in traumatic brain injury and Alzheimer's disease. During traumatic brain injury, intracranial pressures may reach 100 mmHg (13.3 kPa), which is accompanied by patient deaths. This is unsurprising because the pressure exceeds that necessary to rupture cell membranes (~12 kPa); this also highlights the importance of early intervention. Heightened intracranial pressures coupled with the LPP will disrupt membranes. *ABCA1* and *APOE4* polymorphisms predispose individuals to negative outcomes in traumatic brain injury (Table 1). Susceptibility to Alzheimer's disease is associated with *ABCA1, APOE4, GST*, and *CYP46A1* polymorphisms, which may share mechanistic steps with glaucoma. This suggests that pore formation may be a signal event in Alzheimer's disease.

**Table 1:** Polygenic diseases correlating with ABCA1, ApoE4, Glutathione S-Transferase, and CYP46A1 Polymorphisms.

	**Disease susceptibility**			
**Disease**	**ABCA1**	**ApoE4**	**Glutathione S-transferase**	**CYP46A1**
Glaucoma	Mabuchi et al., 2011	Liao et al., 2014	Huang et al., 2013	Fourgeux et al., 2009
Traumatic brain injury	Loane et al., 2011	Houlden and Greenwood, 2006	Al Nimer et al., 2013	
Alzheimer's disease	Koldamova et al., 2010	Schellenberg et al., 2000	Jafarian et al., 2018	Kölsch et al., 2002
Macular degeneration	Fritsche et al., 2016	See text	Güven et al., 2011	Fourgeux et al., 2012

A large GWAS suggests that *ABCA1* polymorphisms are risk factors in age-related macular degeneration (Fritsche et al., 2016). Zareparsi et al. (2004) has shown that *APOE4* is protective in AMD; in contrast, *APOE4* correlates with disease susceptibility in glaucoma, traumatic brain injury, and Alzheimer's disease. Although the mechanism is uncertain, the difference may be due to differences in lipid management of target tissues (Gülcan et al., 1993). Moreover, the *APOE2* polymorphism may contribute to the risk of macular degeneration (McKay et al., 2011); this may be due to APOE2's dysregulation of cholesterol trafficking due to APOE2's poor binding affinity to LDL receptors (Johnson et al., 2014).

## Outlook

Although numerous genetic polymorphisms have been correlated with glaucoma, an understanding of the roles of these genes in disease has been lacking. The mechanical-chemical coupling hypothesis unifies the mechanical stress of heightened IOP and the degradation of surface compressive strength by oxidatively damaged lipids, which lead to membrane destabilization and pore formation. The polygenic nature of POAG is a consequence of the multiple gene products influencing membrane surface tension and repair contributing to membrane stability.

Importantly, the hypothesis and its corollaries can be experimentally tested using, for example, technologies I have previously reported (Francis et al., 1988; Kindzelskii and Petty, 1999) in combination with microscope pressure chambers. Moreover, a new generation of insights should be gained by combining physical and genetic approaches. For example, the theory predicts that kinetic studies of rupture closing using mutant myocilin-expressing cells will exhibit long open times in comparison to controls as these cells are expected to have difficulty in mustering a sufficient number of vesicles to repair membrane damage. In contrast, small pores whose closing is driven by thermodynamic principles should not be sensitive to myocilin expression. It should also be possible to use imaging to detect Ld domains in neuronal cells. Finally, it may be possible to quantitatively measure changes in compressive strength and Young's modulus of neuronal cells in the presence of oxidative events.

In the absence of a known mechanism, it has not been possible to develop new drugs to directly treat retinal damage in glaucoma. *In vitro* pore formation tests, as described in the preceding paragraph, could be used as a screening assay to search for new glaucoma drugs. As damaged lipids may accumulate over time, it will be important to reduce the formation of oxidized lipid products to levels that could be managed by deficient lipid disposal machinery. As DHA supplementation has been reported to reduce the rates of cognitive decline in Alzheimer's disease (Zhang et al., 2016), this may be useful in glaucoma. α-Lipoic acid may also be useful in limiting RGC damage (Inman et al., 2013), which is reasonable because its redox moiety is accessible to the membrane interior. Unfortunately, bioavailability in the retina is a serious issue. Another approach is to minimize pore activity. This could be performed by blocking the pore, increasing the pore's line tension, and/or reducing the RGC's surface tension. Finally, as cells have evolved repair pathways, augmenting these might be useful in designing next-generation pharmaceuticals.

Equation (1) asserts that IOP reduction should benefit patients, and that oxidative reductions in compressive strength must be offset by reductions in IOP to maintain membrane stability. Clinical trials show that a POAG patient's benefit is proportional to the IOP drop (Weinreb and Khaw, 2004). Importantly, drugs that lower IOP in high tension glaucoma are also beneficial in normal tension glaucoma (Collaborative Normal-Tension Glaucoma Study Group, 1998). As pressure reduction is important and independent of its initial IOP value, IOP reduction could be extended to other normal tension retinal diseases. Due to similarities in genetic polymorphisms (Table 1), macular degeneration may benefit from reductions in IOP. Moreover, drugs affecting pore closing should also be helpful in minimizing cell damage in multiple diseases, including macular degeneration, traumatic brain injury, and Alzheimer's disease. The selection of reagents specifically increasing or decreasing surface tension will depend upon their influence on compressive strength vs. pore closing ability. Further biophysical tests and quantitative parameterization is essential, and should enable in-depth computational modeling and innovations in drug development.

POAG is a primarily a disease of membrane repair, both at the level of individual lipid molecules and at the level of plasma membrane/endomembrane trafficking. Ultimately, knowledge of a patient's genetic background could be used to select drugs that improve lipid management or vesicle trafficking to improve patient outcomes.

## Author contributions

The author confirms being the sole contributor of this work and approved it for publication.

### Conflict of interest statement

The author declares that the research was conducted in the absence of any commercial or financial relationships that could be construed as a potential conflict of interest.

## Footnotes

^1^Line tension is the free energy necessary to create the edge or boundary between two phases.

^2^Compressive strength is the ability to resist a load whereas tensile strength is the resistance to being pulled apart.
